# Amino acid changes accumulated in the fusion protein allow neuropathogenic measles viruses to use a broad repertoire of host factors for cell fusion triggering

**DOI:** 10.1128/jvi.02307-24

**Published:** 2025-04-07

**Authors:** Yuichi Hirai, Ryuichi Takemoto, Yusuke Yanagi, Yuta Shirogane

**Affiliations:** 1 Department of Virology, Faculty of Medicine, Kyushu University 12923, Fukuoka, Japan; 2 Department of Molecular Virology, Graduate School of Medical and Dental Sciences, Institute of Science Tokyo, Tokyo, Japan; 3 Department of Pediatrics, Graduate School of Medical Sciences, Kyushu University 12923, Fukuoka, Japan; University Medical Center Freiburg, Freiburg, Germany

**Keywords:** paramyxovirus, measles virus, membrane fusion, cell fusion, SSPE, virus evolution, *cis*-acting fusion triggering, viral envelope, hemagglutinin, fusion protein

## Abstract

Measles virus (MeV), an enveloped RNA virus belonging to the genus *Morbillivirus* of the family *Paramyxoviridae*, is the causative agent of measles, an acute febrile illness with skin rash. MeV has two types of envelope glycoproteins: the hemagglutinin (H) and fusion (F) protein. The H protein initially binds to its receptors, signaling lymphocytic activation molecule family member 1 (SLAMF1) and nectin-4, triggering conformational changes in the F protein that result in virus-to-cell or cell-to-cell membrane fusion. MeV may persist in the brain, which does not express SLAMF1 and nectin-4, leading to subacute sclerosing panencephalitis (SSPE) several years after acute infection. Recently, we have reported that MeV isolates from SSPE patients have hyperfusogenic amino acid changes (e.g., T461I) in the F protein, which enable MeV to spread in the brain by using cell adhesion molecule 1 (CADM1) and CADM2 as *cis*-acting fusion-triggering molecules. However, F proteins of SSPE isolates, such as the Patient B and OSA-3/Bs/B strains, carry additional changes besides T461I. In this study, we show that specific combinations of amino acid changes in the F proteins from SSPE isolates enable the induction of membrane fusion without CADM1/2 expression. We further demonstrate that these cumulative changes in the F protein allow the virus to exploit other fusion-triggering molecules than CADM1/2. These changes also promote efficient neuronal cell fusion. Our findings suggest that cumulative changes in the F protein may broaden the range of host factors capable of triggering cell fusion, facilitating MeV spread in the brain of SSPE patients.

**IMPORTANCE:**

Subacute sclerosing panencephalitis (SSPE) is a fatal disease caused by persistent infection of measles virus (MeV) in the brain. There is no effective therapy for the disease. MeV isolates from SSPE patients accumulate multiple amino acid changes in the F protein, including hyperfusogenic changes such as the T461I substitution, which allow MeV to spread in the brain by utilizing cell adhesion molecule 1 (CADM1) and CADM2 as *cis*-acting fusion-triggering molecules. In this study, we show that F proteins of SSPE isolates harboring additional changes besides T461I can induce membrane fusion independently of CADM1 and CADM2. The data also indicate that cumulative changes in the F protein may enable MeV to use other fusion-triggering host molecules than CADM1 and CADM2, facilitating its spread in the brain of SSPE patients. The findings deepen our understanding of the molecular mechanism underlying MeV neuropathogenicity in SSPE.

## INTRODUCTION

Measles is a highly contagious disease characterized by high fever, respiratory symptoms, conjunctivitis, and maculopapular rash (1, 2). The causative agent of measles is measles virus (MeV), an enveloped virus with a non-segmented negative stranded RNA genome belonging to the genus *Morbillivirus* of the family *Paramyxoviridae*. MeV has two types of glycoproteins on its envelope, the hemagglutinin (H) and fusion (F) protein. These proteins cooperatively mediate membrane fusion required for MeV entry into the target cell and direct cell-to-cell spread (2). During membrane fusion, the H protein initially binds to its receptors, signaling lymphocytic activation molecule family member 1 (SLAMF1) on immune cells (3) and nectin-4 on epithelial cells (4, 5). The F protein then undergoes conformational changes from the prefusion form to the postfusion form, leading to membrane fusion (6).

MeV may persist in the human brain, causing subacute sclerosing panencephalitis (SSPE) several years after acute infection (2). SSPE is a fatal progressive neurological disorder that manifests as behavioral changes, cognitive deterioration, myoclonic jerks, and mental impairment (7). The incidence rate is estimated to be 6.5–11 cases per 100,000 measles cases (7). There is currently no effective therapy for SSPE (8).

Wild-type (WT) MeV strains isolated from patients with acute measles do not have the ability to spread in the brain, which lacks expression of SLAMF1 and nectin-4 (9, 10). However, in the brain of SSPE patients, MeV genomes spread between neurons, presumably through cell fusion at neuronal synapses (11–14). We and others have recently reported that certain amino acid changes (e.g., T461I) in the F protein of MeV strains isolated from SSPE patients allow them to spread in the central nervous system (13, 15–27). These changes in the F protein are thought to lower the threshold of activation energy required for the F protein to undergo conformational changes, allowing even weak interactions of the H protein with host molecules to induce membrane fusion (15, 28). We have also reported that recombinant MeVs with those hyperfusogenic mutations in the F gene can use cell adhesion molecule 1 (CADM1, also known as IGSF4A, Necl-2, and SynCAM1) and CADM2 (also known as IGSF4D, Necl-3, and SynCAM2) as “*cis*-acting receptor-mimicking molecules” in neurons (29–31). CADM1 and CADM2 interact in *cis* with the H protein on the same cell membrane to trigger the conformational change of the mutant F protein, leading to cell-to-cell fusion and allowing direct MeV genome transfer between neurons.

However, the F proteins from MeV SSPE isolates carry various functionally unknown changes in addition to those identified as hyperfusogenic changes. In this study, we show that some SSPE-derived F proteins can mediate cell-to-cell fusion even in the absence of CADM1 and CADM2 expression. By reversing respective mutations in the F gene of MeV SSPE isolates to the WT sequence, we identified specific combinations of mutations that enable CADM1/2-independent membrane fusion. When such mutant F proteins are expressed with the H protein, membrane fusion can be induced by other host molecules than CADM1/2. We also show that these mutant F proteins enhance cell-to-cell fusion between primary neurons.

## RESULTS

### SSPE-derived F proteins mediate CADM1/2-independent membrane fusion

**Fig 1:**
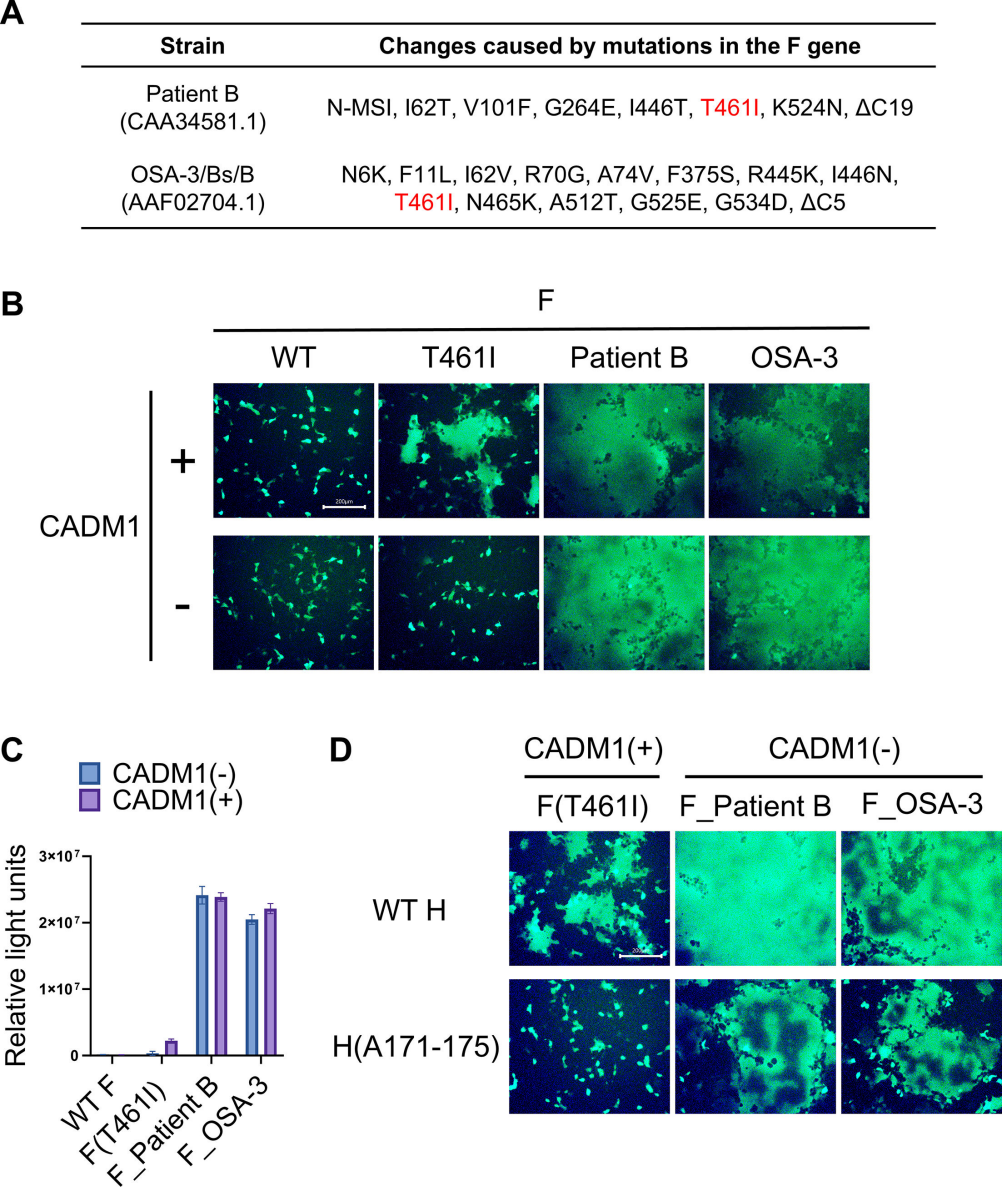
SSPE-derived F proteins mediate CADM1/2-independent membrane fusion. (A) Amino acid changes found in the F protein of the Patient B strain (CAA34581.1, GenBank) and the OSA-3/Bs/B strain (AAF02704.1, GenBank). N-MSI indicates three amino acids (methionine, serine, and isoleucine) added at the N-terminus. ΔC5 and ΔC19 indicate the deletion of 5 and 19 amino acids at the C-terminus, respectively. (B) The pCA7 plasmids respectively encoding the WT H, MeV F [WT F, F(T461I), F_Patient B, or F_OSA-3], EGFP, and CADM1 or none were transfected into 293FT cells. The cells were observed under a fluorescence microscope 24 hours after transfection. Scale bar, 200 µm. (C) The pCA7 plasmids respectively encoding the WT H, MeV F [WT F, F(T461I), F_Patient B, or F_OSA-3], and CADM1 or none were transfected into cocultured 293FT/DSP1 and 293FT/DSP2 cells. The *Renilla* luciferase activity was analyzed 24 hours after transfection (*n* = 3 biological replicates, means ± SD). (D) The pCA7 plasmids respectively encoding MeV H [WT H or H(A171-175)], MeV F [F(T461I), F_Patient B, or F_OSA-3], EGFP, and CADM1 or none were transfected into 293FT cells. The cells were observed under a fluorescence microscope 24 hours after transfection. Scale bar, 200 µm.

We have previously shown that CADM1 and CADM2 trigger membrane fusion mediated by hyperfusogenic mutant MeV F proteins [e.g., the F protein possessing the T461I substitution, F(T461I)] but not the WT F protein (29, 31). However, F proteins derived from MeV SSPE isolates usually have multiple changes (26). For example, the F proteins from the Patient B and OSA-3/Bs/B strains have amino acid substitutions at several positions and alterations at N- and/or C-termini, in addition to the T461I substitution (Fig. 1A). When transiently expressed together with the H protein in 293FT cells, these F proteins, F_Patient B and F_OSA-3, exhibited enhanced CADM1-dependent fusion activity, as compared with F(T461I) (Fig. 1B) (12). Unexpectedly, F_Patient B and F_OSA-3 induced syncytium formation even without transient expression of CADM1, unlike F(T461I) (Fig. 1B).

The levels of membrane fusion mediated by these different F proteins were also quantified by using the dual split protein (DSP) assay system (28, 32–34). In this assay, a pair of chimeric reporter proteins, DSP1 and DSP2, each consisting of the split *Renilla* luciferase and split green fluorescent protein (GFP), are stably expressed in 293FT cells, respectively (293FT/DSP1 and 293FT/DSP2 cells). When cell fusion is induced between 293FT/DSP1 and 293FT/DSP2 cells, the *Renilla* luciferase and GFP activities are restored by the association of DSP1 and DSP2. 293FT/DSP1 and 293FT/DSP2 cells were cocultured and then transfected with plasmids respectively encoding the H protein and one of the F proteins (WT F, F(T461I), F_Patient B, or F_OSA-3) and a plasmid encoding CADM1 or none. F_Patient B and F_OSA-3 induced cell fusion even without CADM1 expression, whereas WT F and F(T461I) did not (Fig. 1C). Thus, these quantitative data also indicate that F_Patient B and F_OSA-3 can mediate membrane fusion independently of CADM1.

The low level of CADM1 may be endogenously expressed in 293FT cells (29). To exclude the possibility that the endogenous CADM1 in 293FT cells contributes to membrane fusion mediated by the F_Patient B and F_OSA-3, we examined whether cell fusion is observed when the “CADM-blind” H protein, H(A171-175), was expressed with these F proteins. H(A171-175) has alanine substitutions at the amino acid residues 171 to 175 of the H protein, which abolish its ability to use CADM1/2 as *cis*-acting receptor-mimicking molecules (30). Syncytium formation was not observed when H(A171-175) was expressed with F(T461I) and CADM1 in 293FT cells (Fig. 1D). However, F_Patient B and F_OSA-3 still caused cell fusion even when expressed with H(A171-175) (Fig. 1D). These results support that F_Patient B and F_OSA-3 can mediate membrane fusion independently of CADM1.

### CADM1/2-independent membrane fusion is enabled by the combined G264E and T461I substitutions in F_Patient B

**Fig 2:**
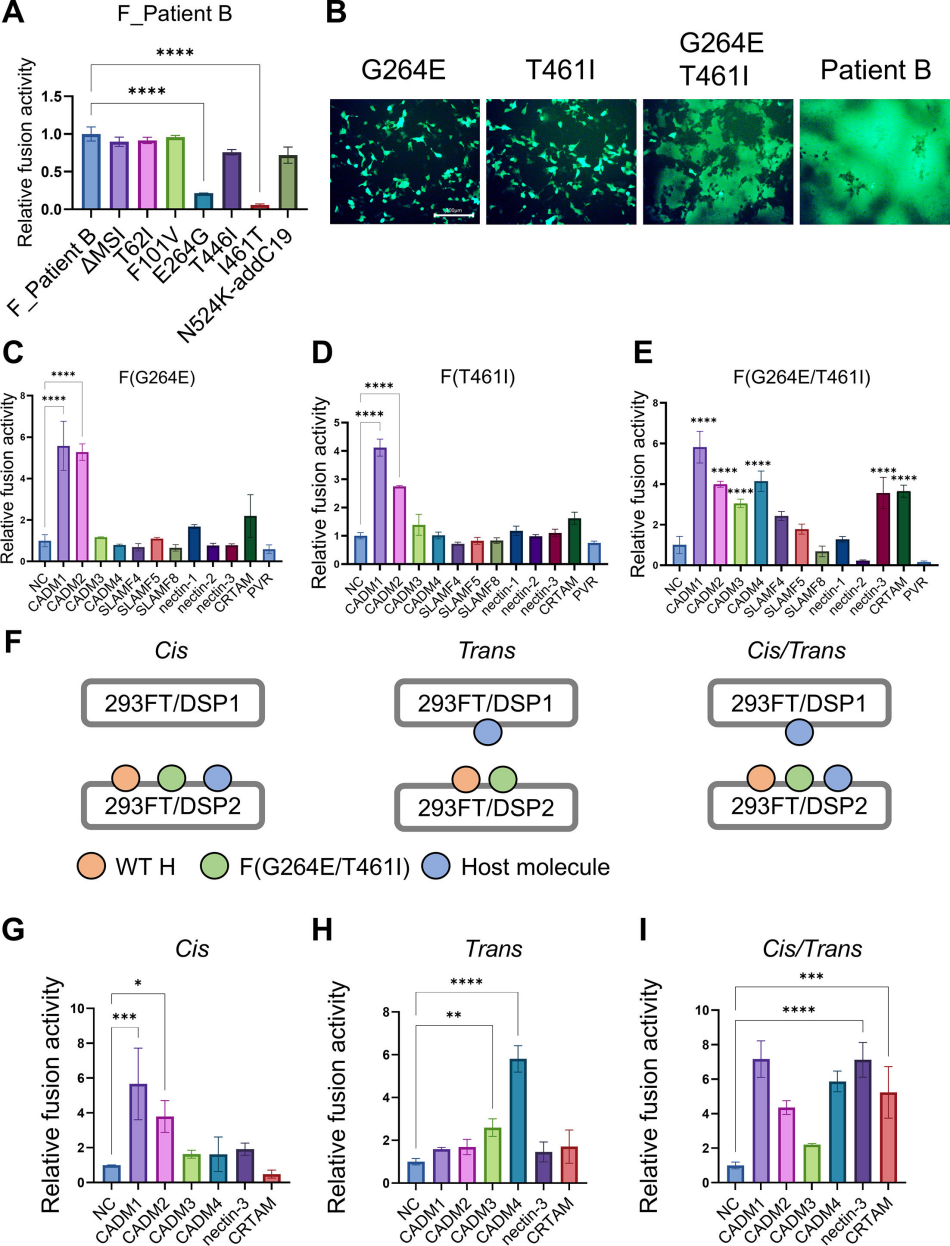
The combined G264E and T461I substitutions in the F protein enable CADM1/2-independent membrane fusion. (A) The pCA7 plasmids respectively encoding the WT H and F_Patient B or that with reverted mutation(s) (ΔMSI, T62I, F101V, E264G, T446I, I461T, or N524K-addC19) were transfected into cocultured 293FT/DSP1 and 293FT/DSP2 cells. ΔMSI indicates the deletion of the three amino acids (methionine, serine, and isoleucine) at the N-terminus of F_Patient B. N524K-addC19 indicates the reverted mutation at residue 524 (N524K) and the 19 amino acid insertion at the C-terminus. The *Renilla* luciferase was analyzed 24 hours after transfection (*n* = 3 biological replicates, means ± SD). The fusion activity of F_Patient B was set to 1.0. Statistical differences were assessed with one-way analysis of variance: ****, *P* < 0.0001. (B) The pCA7 plasmids respectively encoding the WT H, MeV F [F(G264E), F(T461I), F(G264E/T461I), or F_Patient B], and EGFP were transfected into 293FT cells. The cells were observed under a fluorescence microscope 24 hours after transfection. Scale bar, 200 µm. (C, D, and E) The pCA7 plasmids respectively encoding the WT H, MeV F [F(G264E) (C), F(T461I) (D), or F(G264E/T461I) (E)], and one of the host molecules (CADM1, CADM2, CADM3, CADM4, SLAMF4, SLAMF5, SLAMF8, nectin-1, nectin-2, nectin-3, CRTAM, PVR, or none [NC: pCA7 alone as a negative control]) were transfected into cocultured 293FT/DSP1 and 293FT/DSP2 cells. The *Renilla* luciferase activity was analyzed 24 hours after transfection (*n* = 3 biological replicates, means ± SD). The fusion activities of respective NC samples were set to 1.0. Statistical differences were assessed with one-way analysis of variance: ****, *P* < 0.0001. (F) Schematic diagrams of the DSP assay performed in the following experiments. The left, middle, and right diagrams correspond to G, H, and I, respectively. (G) Evaluation of *cis*-acting fusion triggering functions of host molecules. 293FT/DSP2 cells were transfected with pCA7 plasmids respectively encoding the WT H, F(G264E/T461I), and one of the host molecules (CADM1, CADM2, CADM3, CADM4, nectin-3, CRTAM, or none). The cells were mixed with 293FT/DSP1 cells transfected with the empty pCA7 plasmid. The *Renilla* luciferase activity was analyzed 24 hours after transfection (*n* = 3 biological replicates, means ± SD). The fusion activity of the NC sample was set to 1.0. Statistical differences were assessed with one-way analysis of variance: *, *P* < 0.05, ***, *P* < 0.001. (H) Evaluation of *trans*-acting fusion triggering functions of host molecules. 293FT/DSP2 cells were transfected with pCA7 plasmids respectively encoding the WT H and F(G264E/T461I). The cells were mixed with 293FT/DSP1 cells transfected with pCA7 plasmids encoding one of the host molecules (CADM1, CADM2, CADM3, CADM4, nectin-3, CRTAM, or none). The *Renilla* luciferase activity was analyzed 24 hours after transfection (*n* = 3 biological replicates, means ± SD). The fusion activity of the NC sample was set to 1.0. Statistical differences were assessed with one-way analysis of variance: **, *P* < 0.01, ****, *P* < 0.0001. (I) Evaluation of fusion triggering functions of host molecules when they were expressed in both *cis* and *trans*. 293FT/DSP2 cells were transfected with pCA7 plasmids respectively encoding the WT H, F(G264E/T461I), and one of the host molecules (CADM1, CADM2, CADM3, CADM4, nectin-3, CRTAM, or none). The cells were mixed with 293FT/DSP1 cells transfected with pCA7 plasmids encoding the corresponding host molecule (CADM1, CADM2, CADM3, CADM4, nectin-3, CRTAM, or none) (*n* = 3 biological replicates, means ± SD). The fusion activity of the NC sample was set to 1.0. Statistical differences were assessed with one-way analysis of variance: ***, *P* < 0.001, ****, *P* < 0.0001.

We investigated which amino acid changes found in F_Patient B were responsible for CADM1/2-independent membrane fusion by reverting each of the amino acid changes back to the WT F sequence. The levels of membrane fusion mediated by these reverted F proteins were quantified by using the DSP assay. When the E264G or I461T substitution was introduced into F_patient B, the level of membrane fusion was significantly decreased (Fig. 2A). The result suggests that the G264E and T461I substitutions are important for F_Patient B to induce CADM1/2-independent membrane fusion. Indeed, the F protein with both substitutions, F(G264E/T461I), induced membrane fusion in the absence of CADM1 expression in 293FT cells, whereas F(G264E) and F(T461I) did not (Fig. 2B).

We next examined whether host molecules other than CADM1/2 allow membrane fusion mediated by F(G264E/T461I). Host molecules expressed in the human brain and belonging to the SLAM, nectin, and CADM families (which contain the known MeV receptors and receptor-mimicking molecules) were tested for their ability to trigger CADM1/2-independent membrane fusion. Consistent with our previous findings (29), only CADM1 and CADM2 allowed F(G264E)- or F(T461I)-mediated membrane fusion (Fig. 2C and D). In contrast, F(G264E/T461I)-mediated membrane fusion was triggered by CADM3, CADM4, nectin-3, and CRTAM) in addition to CADM1/2 (Fig. 2E). The result indicates that the accumulation of hyperfusogenic changes in the F protein allows MeV to induce cell fusion by using host molecules other than CADM1/2.

We then investigated whether CADM3, CADM4, nectin-3, and CRTAM act in *cis* (in cells also expressing MeV envelope proteins) or in *trans* (in cells other than those expressing MeV envelope proteins, like the usual receptors). These host molecules were expressed in *cis* or in *trans* with respect to the H and F proteins in the DSP assay (Fig. 2F). When expressed in *cis*, CADM1 and CADM2 allowed membrane fusion mediated by F(G264E/T461I), whereas the other host molecules examined did not (Fig. 2G). When expressed in *trans*, CADM3 and CADM4 triggered F(G264E/T461I)-mediated membrane fusion (Fig. 2H). Nectin-3 and CRTAM triggered membrane fusion only when expressed simultaneously in *cis* and in *trans* (Fig. 2I). These data suggest that cumulative amino acid changes in the F protein allow MeV to use fusion-triggering host molecules that act in three different ways, in *cis*, in *trans*, and in both *cis* and *trans*.

### CADM1/2-independent membrane fusion is enabled by the combination of F375S, I446N, and T461I substitutions in F_OSA-3

**Fig 3:**
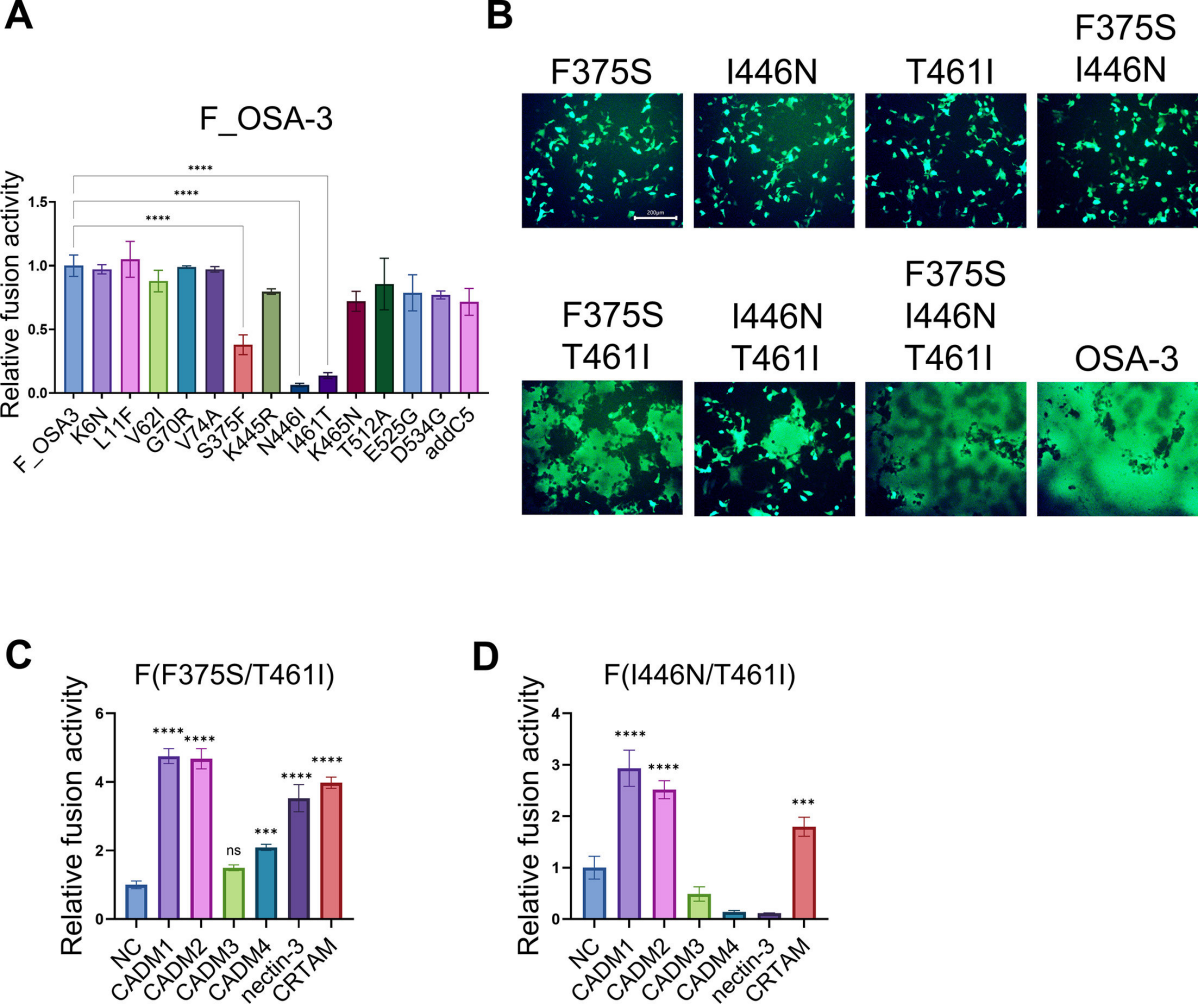
Certain combinations of the F375S, I446N, and T461I substitutions in the F protein enable CADM1/2-independent membrane fusion. (A) The pCA7 plasmids respectively encoding the WT H and F_OSA-3 or that with reverted mutation(s) (K6N, L11F, V62I, G70R, V74A, S375F, K445R, N446I, I461T, K465N, T512A, E525G, D534G, or addC5) were transfected into cocultured 293FT/DSP1 and 293FT/DSP2 cells. addC5 indicates the five amino acid insertion at the C-terminus of F_OSA-3. The *Renilla* luciferase activity was analyzed 24 hours after transfection (*n* = 3 biological replicates, means ± SD). The fusion activity of F_OSA-3 was set to 1.0. Statistical differences were assessed with one-way analysis of variance: ****, *P* < 0.0001. (B) The pCA7 plasmids respectively encoding the WT H, MeV F [F(F375S), F(I446N), F(T461I), F(F375S/I446N), F(F375S/T461I), F(I446N/T461I), F(F375S/I446N/T461I), or F_OSA-3], and EGFP were transfected into 293FT cells. The cells were observed under a fluorescence microscope 24 hours after transfection. Scale bar, 200 µm. (C and D) The plasmids respectively encoding the WT H, MeV F [F(F375S/T461I) (C) or F(I446N/T461I) (D)], and one of the host molecules (CADM1, CADM2, CADM3, CADM4, nectin-3, CRTAM, or none [NC]) were transfected into cocultured 293FT/DSP1 and 293FT/DSP2 cells (*n* = 3 biological replicates, means ± SD). The fusion activity of the NC samples was set to 1.0. Statistical differences were assessed with one-way analysis of variance: ns, not significant, ***, *P* < 0.001, ****, *P* < 0.0001.

We also examined F_OSA-3 as we did for F_Patient B. Each of the amino acid changes in F_OSA-3 was reverted to the WT F protein sequence. When the S375F, N446I, or I461T substitution was introduced into F_OSA-3, the level of membrane fusion was significantly reduced (Fig. 3A). The data suggest that the F375S, I446N, and T461I substitutions are responsible for the ability of F_OSA-3 to induce CADM1/2-independent membrane fusion. When expressed with the H protein, F(F375S/T461I), F(I446N/T461I), and F(F375S/I446N/T461I) induced CADM1-independent membrane fusion in 293FT cells, whereas F(F375S), F(I446N), F(T461I), and F(F375S/I446N) failed to do so (Fig. 3B). Thus, the accumulation of mutations in the F gene enables the OSA-3/Bs/B strain to induce CADM1/2-independent membrane fusion.

We studied whether CADM3, CADM4, nectin-3, and CRTAM could allow membrane fusion mediated by F(F375S/T461I) and F(I446N/T461I), like that mediated by F(G264E/T461I). F(F375S/I446N/T461I) was not examined because the transfected cells fused in the entire field even without the expression of host molecules, making it difficult to evaluate whether host candidate molecules can trigger membrane fusion (data not shown).

The result of the DSP assay shows that CADM1, CADM2, CADM4, nectin-3, and CRTAM, but not CADM3, allowed F(F375S/T461I)-mediated membrane fusion (Fig. 3C). On the other hand, CADM1, CADM2, and CRTAM allowed membrane fusion mediated by F(I446N/T461I), while CADM3, CADM4, and nectin-3 did not (Fig. 3D). These results suggest that different repertoires of host molecules may trigger membrane fusion mediated by different mutant F proteins during MeV persistent infection.

### The I446N substitution in the F protein has an inhibitory effect on CADM1/2-independent membrane fusion in CHO cells

**Fig 4:**
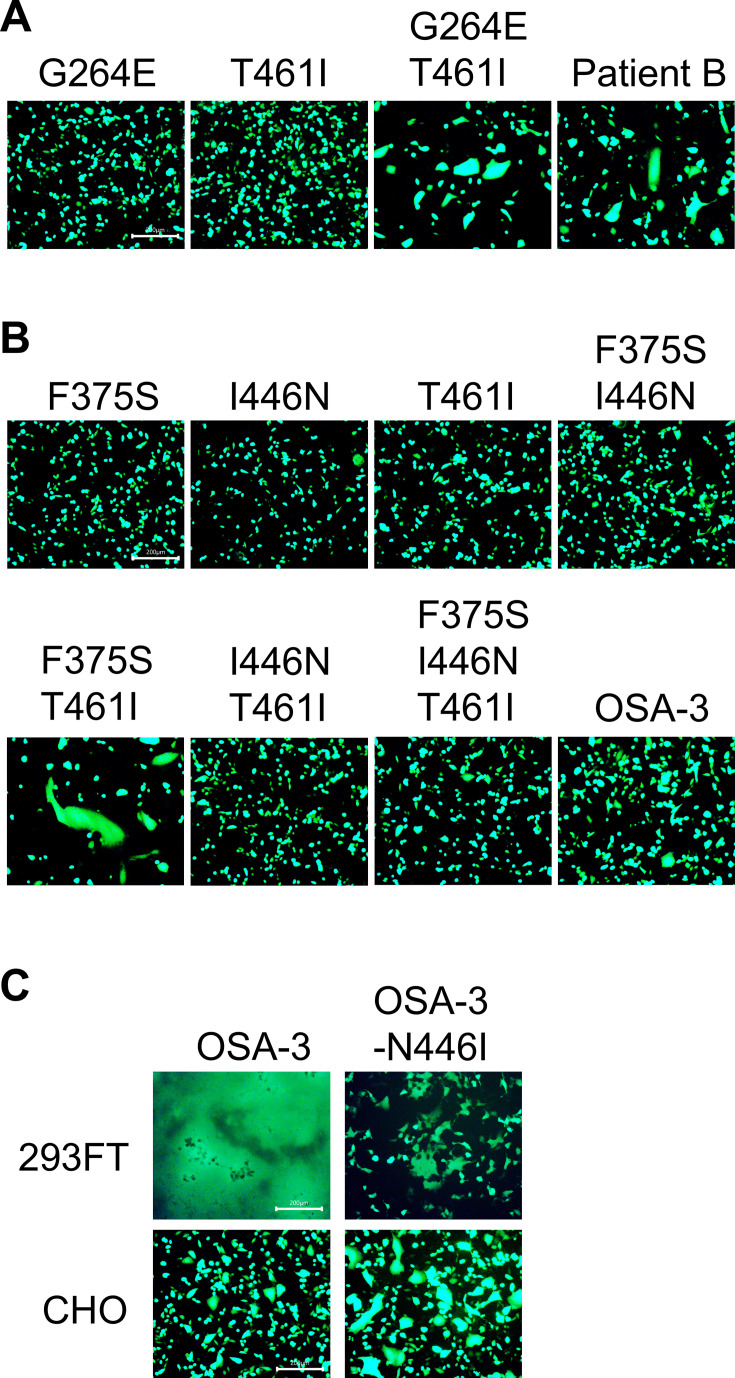
The I446N substitution in the F protein has an inhibitory effect on membrane fusion in CHO cells. (A) The pCA7 plasmids respectively encoding the WT H, MeV F [F(G264E), F(T461I), F(G264E/T461I), or Patient B], and EGFP were transfected into CHO cells. The cells were observed under a fluorescence microscope 30 hours after transfection. Scale bar, 200 µm. (B) The pCA7 plasmids respectively encoding the WT H, MeV F [F(F375S), F(I446N), F(T461I), F(F375S/I446N), F(F375S/T461I), F(I446N/T461I), F(F375S/I446N/T461I), or F_OSA-3], and EGFP were transfected into CHO cells. The cells were observed under a fluorescence microscope 30 hours after transfection. Scale bar, 200 µm. (C) The pCA7 plasmids respectively encoding the WT H, MeV F (F_OSA-3 or F_OSA-3-N446I), and EGFP were transfected into 293FT and CHO cells, respectively. The 293FT and CHO cells were observed under a fluorescence microscope 24 and 30 hours after transfection, respectively. Scale bar, 200 µm.

All membrane fusion experiments described above were performed using a human cell line 293FT. Therefore, we also used another cell line, CHO. When expressed with the H protein, F_Patient B and F(G264E/T461I), but not F(G264E) and F(T461I), induced syncytia in CHO cells (Fig. 4A), consistent with the results in 293FT cells (Fig. 2B). On the other hand, the F proteins having different combinations of amino acid changes found in the OSA-3/Bs/B strain exhibited different patterns of syncytium formation in CHO cells (Fig. 4B) from those in 293FT cells (Fig. 3B). Even F_OSA-3 induced only small syncytia in CHO cells. Although F(F375S/T461I) produced apparent syncytia, F(F375S/I446N), F(I446N/T461I), and F(F375S/I446N/T461I) hardly induced cell-to–cell fusion (Fig. 4B). The results suggest that the presence of the I446N substitution in the F protein may have an inhibitory effect on CADM1/2-independent membrane fusion in CHO cells. To test this hypothesis, we produced the plasmid encoding F_OSA-3 with the N446I substitution. F_OSA-3-N446I induced larger syncytia in CHO cells but smaller syncytia in 293FT cells, as compared with F_OSA-3 (Fig. 4C), supporting our hypothesis. These results indicate that mutant F proteins mediate membrane fusion differently depending on host molecules expressed in host cells.

**Fig 5:**
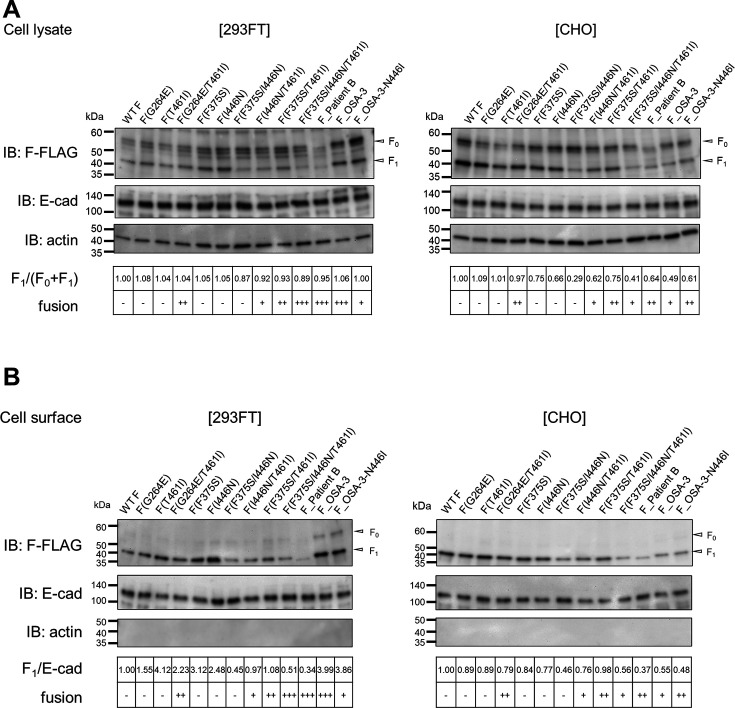
Cell surface biotinylation assay with MeV F proteins used in this study. (A and B) E-cadherin (a cell surface protein control) was expressed together with one of the Flag-tagged MeV F proteins [WT F, F(G264E), F(T461I), F(G264E/T461I), F(F375S), F(I446N), F(F375S/I446N), F(I446N/T461I), F(F375S/T461I), F(F375S/I446N/T461I), F_Patient B, F_OSA-3, or F_OSA-3-N446I] in 293FT and CHO cells. Cell lysates (A) and precipitates of biotinylated cell surface proteins (B) were examined by western blotting using anti-Flag (upper), anti-E-cadherin (middle), and anti-β-actin (bottom) antibodies. Protein band signals were quantified, and the ratios of F_1_ to (F_0_ + F_1_) (A) and those of F_1_ to E-cadherin (B) were calculated. The ratios of the WT F protein were set to 1.00. The levels of CADM1/2-independent fusion activity of the respective F proteins are also shown (- to +++).

We examined the cleavage of the F proteins used in this study, and there was no correlation between their cleavage efficiencies and fusogenicities (Fig. 5A). The cell surface expression levels of the F proteins were also examined by the surface biotinylation assay, and there was no correlation between their cell surface expression levels and fusogenicities (Fig. 5B). Thus, the levels of CADM1/2-independent membrane fusion mediated by the mutant F proteins in this study are likely to reflect their intrinsic fusogenicities in respective host cells.

### Cumulative amino acid changes in F_Patient B and F_OSA-3 promote neuronal spread

**Fig 6:**
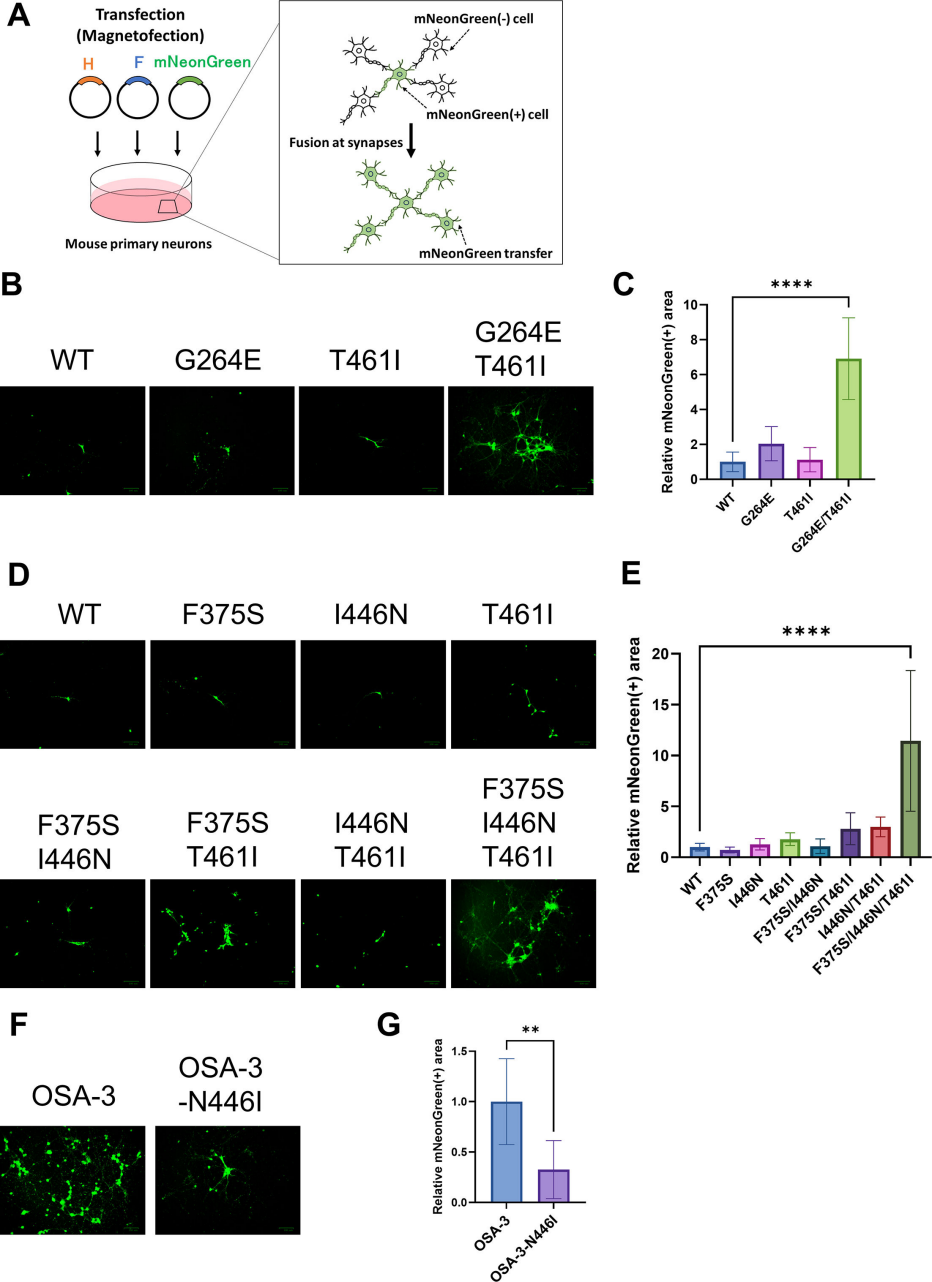
Cumulative amino acid changes found in F_Patient B and F_OSA-3 enhance neuronal cell fusion. (A) A schematic diagram of the fusion-mediated spread assay. (B and C) The pCA7 plasmids respectively encoding the WT H, MeV F [WT F, F(G264E), F(T461I), or F(G264E/T461I)], and mNeonGreen were transfected into mouse primary neurons. The cells were observed under a fluorescence microscope 24 hours after transfection (B). Scale bar, 100 µm. The areas of neurons expressing mNeonGreen were also quantified by using ImageJ software (36) (*n* = 6, means ± SD) (C). Statistical differences were assessed with one-way analysis of variance: ****, *P* < 0.0001. The mNeonGreen-expressing area of the WT F sample was set to 1.0. (D and E) The pCA7 plasmids respectively encoding the WT H, MeV F [WT F, F(F375S), F(I446N), F(T461I), F(F375S/I446N), F(F375S/T461I), F(I446N/T461I), or F(F375S/I446N/T461I)], and mNeonGreen were transfected into mouse primary neurons. The cells were observed under a fluorescence microscope 24 hours after transfection (D). Scale bar, 100 µm. The areas of neurons expressing mNeonGreen were also quantified by using ImageJ software (*n* = 6, means ± SD) (E). The mNeonGreen-expressing area of the WT F sample was set to 1.0. Statistical differences were assessed with one-way analysis of variance: ****, *P* < 0.0001. (F and G) The pCA7 plasmids respectively encoding the WT H, MeV F (F_OSA-3 or F_OSA-3-N446I), and mNeonGreen were transfected into neurons. The cells were observed under a fluorescence microscope 30 hours after transfection (F). Scale bar, 100 µm. The areas of neurons expressing mNeonGreen were also quantified by using ImageJ (*n* = 6, means ± SD) (G). The mNeonGreen-expressing area of the F_OSA-3 sample was set to 1.0. Statistical differences were assessed with an unpaired *t* test: **, *P* < 0.01.

We next examined how amino acid changes in SSPE-derived F proteins affect their fusion activities in neurons, where MeV replicates in SSPE patients (2). To this end, we established the fusion-mediated spread assay using primary neurons. In SSPE patients, MeV genomes are thought to spread transsynaptically between neurons, via MeV glycoprotein-mediated fusion at synapses (11, 14). We transfected mouse primary neurons with pCA7 plasmids respectively encoding the WT H protein, one of the mutant F proteins used in this study, and mNeonGreen, a GFP (35), and observed the cells under a fluorescence microscope (Fig. 6A). When membrane fusion occurs in neurons, mNeonGreen proteins (and possibly transfected plasmids) spread from the initially transfected neurons to neighboring non-transfected neurons (Fig. 6B, D and F). The degree of cell-to–cell fusion between neurons was then quantified by measuring the area of neurons expressing mNeonGreen (Fig. 6C, E and G).

When expressed with the H protein in mouse primary neurons, F(G264E/T461I) possessing amino acid changes found in the Patient B strain mediated increased spread of mNeonGreen as compared with the WT F (Fig. 6B and C). Among the mutant F proteins possessing amino acid changes found in the OSA-3/Bs/B strain, F(F375S/I446N/T461I) allowed the most increased spread of mNeonGreen as compared with the WT F (Fig. 6D and E). Furthermore, the I446N substitution was found to be important in neuronal spread of the OSA-3/Bs/B strain, as F_OSA3-N446I could allow less spread of mNeonGreen than F_OSA-3 (Fig. 6F and G). All these results with the fusion-mediated spread assay using primary neurons are consistent with the fusion assay data in 293FT cells.

## DISCUSSION

This study showed that cumulative F gene mutations found in MeV isolates from SSPE patients allow them to induce membrane fusion in a CADM1/2-independent manner. The combined G264E and T461I substitutions in F_Patient B and certain combinations of the F375S, I446N, and T461I substitutions in F_OSA-3 were found to be sufficient for the induction of membrane fusion without CADM1/2 expression (Fig. 2A and B, and 3A and B). Such amino acid changes in the F protein enabled membrane fusion to be triggered by various repertoires of host molecules besides CADM1/2 (Fig. 2E, and 3C and D). Interestingly, CADM3 and CADM4 acted in *trans* with respect to the H and F proteins to induce F(G264E/T461I)-mediated membrane fusion (Fig. 2H). In contrast, nectin-3 and CRTAM induced the membrane fusion when they were expressed in both *trans* and *cis* (Fig. 2I).

We have previously reported that hyperfusogenic amino acid substitutions in the F protein allow MeV to use host molecules which weakly interact with the H protein in *cis* or *trans* for fusion triggering, presumably by decreasing the stability of the prefusion form of the F protein and lowering the activation energy level required to induce its conformational changes (28). Such amino acid substitutions are thought to be clustered in three specific regions in the F protein: site I, site II, and site III (6). Indeed, the substitutions reported in this study are located in these sites: G264E in site II, F375S in site I, and I446N and T461I in site III. The data suggest that the accumulation of the mutations in the F gene further decreases the stability of the F protein and confers on MeV the ability to use more non-canonical host molecules which weakly interact with the H protein in *cis* and/or in *trans*. It should be noted that we only examined the host molecules expressed in the human brain and belonging to the SLAM, nectin, and CADM families. Thus, other unexamined host molecules may also trigger membrane fusion mediated by these mutant F proteins.

Why do some host molecules, nectin-3 and CRTAM, need to be expressed both in *cis* and *trans* with respect to the H protein for triggering membrane fusion? We currently hypothesize two possible mechanisms to explain this phenomenon. First, the interaction of host molecules with the H protein from different orientations (*cis* and *trans*) may facilitate the conformational changes of the H protein, which transmit the fusion-triggering signal to the F protein. Second, the *trans*-homophilic interaction of nectin-3 and CRTAM (37, 38) may generate a new interface and allow for efficient interaction with the H protein. Host molecules acting only in *cis* or *trans* may efficiently interact with the H protein even if they are not simultaneously expressed on the opposite side of the membrane.

We found that the I446N substitution in F_OSA-3 increases fusion activity in 293FT cells but inhibits it in CHO cells, although the mechanism underlying this finding is currently unknown. Thus, it is important to evaluate the fusion activity of the F protein in the cell type of interest, neurons in this case, as mutant F proteins may exhibit different phenotypes depending on cell types. In this study, we examined fusion activity by transiently expressing the H protein, the F protein, and the mNeonGreen protein in neurons. This fusion-mediated spread assay system using neurons allows evaluation of MeV neuronal spread even when mutant virus particles are difficult to recover due to their low stability. Using this system, we successfully demonstrated that combined G264E and T461I substitutions in F_Patient B and certain combinations of the F375S, I446N, and T461I substitutions in F_OSA-3 result in greater fusion activity in neurons, compared to F(T461I). This system might be a useful tool for evaluating the pathogenesis of other viruses that infect neurons, such as herpes simplex virus type 1, Hendra virus, and Nipah virus.

Since CADM1 and CADM2 are abundantly expressed in the central nervous system (29), the significance of MeV acquiring the ability to use other fusion-triggering molecules than CADM1/2 is intriguing. Indeed, recombinant MeVs with a single amino acid change (e.g., T461I) in the F protein, which can use CADM1/2 but no other host molecules as fusion inducers (29), spread in neurons and cause neuropathogenicity in mice and hamsters (11, 22, 26). These observations suggest that CADM1/2 play an important role at least for the initial MeV adaptation in the brain. However, subsequent cumulative mutations in the F gene allow more efficient fusion in neurons than single mutations (Fig. 6). The ability to use additional host fusion-triggering molecules during persistence likely contributes to maximizing the efficiency of MeV spread in the brain of SSPE patients. Thus, cumulative mutations in the F gene allow MeV to further adapt to the brain, causing strong neuropathogenicity and ultimately claiming the lives of SSPE patients.

## MATERIALS AND METHODS

### Cells

293FT cells were maintained in Dulbecco’s modified Eagle medium (DMEM; Fujifilm Wako Pure Chemical Corporation) supplemented with 10% fetal bovine serum (FBS). The 293FT cell line is a derivative of 293T and was obtained from Invitrogen. The 293FT cells stably expressing DSP1 and DSP2 (32–34), kindly provided by Z. Matsuda, the University of Tokyo, were maintained in DMEM supplemented with 10% FBS and 1-µg/mL puromycin (Invivogen). CHO cells were maintained in RPMI medium (Wako) supplemented with 10% FBS. Mouse primary neurons were isolated from the hippocampus of C57BL/6 mouse at embryonic day 17 and cultured according to the protocol described previously (29). All animal experiments were reviewed by the Institutional Animal Care and Use Committee and carried out according to the Guidelines for Animal Experiments of Institute of Science Tokyo, Tokyo, Japan.

### Plasmids

The eukaryotic expression vector pCA7 (39) is a derivative of pCAGGS (40). The pCA7 plasmids respectively encoding MeV H (the Ichinose-B (IC-B) strain), MeV F protein (the IC-B strain), and human CADM1, CADM2, CADM3, CADM4, SLAMF4, SLAMF5, SLAMF8, nectin-1, nectin-2, nectin-3, CRTAM, PVR, E-cadherin, and enhanced green fluorescent protein (EGFP) were described previously (12, 26, 29, 41). For Western blot, the FLAG tag (DYDDDDK) sequences were fused to the C-termini of the WT and mutant F proteins. The plasmid encoding mNeonGreen was purchased from VectorBuilder under the following vector ID number: VB900122-0530ege (mNeonGreen; accession no. KC295282). DNA fragment encoding mNeonGreen was PCR amplified and inserted into pCA7 predigested with EcoRI and NotI.

### Fusion assay

293FT cells cultured in 24-well plates were transfected with different combinations of pCA7 plasmids respectively encoding one of MeV H proteins (WT H or H(A171-175)), one of MeV F proteins used in this study, EGFP, and CADM1 or none, using Lipofectamine LTX (Thermo Fisher Scientific). The cells were observed under a fluorescence microscope 24 hours after transfection. CHO cells cultured in 24-well plates were transfected with different combinations of pCA7 plasmids respectively encoding WT H protein, one of the MeV F proteins used in this study, and EGFP using Lipofectamine LTX (Thermo Fisher Scientific). The cells were observed under a fluorescence microscope 30 hours after transfection.

### DSP assay

DSP1 and DSP2 are split proteins of the *Renilla* luciferase and GFP. They become functional when reassociated with each other after 293FT cells stably expressing DSP1 (293FT/DSP1) and DSP2 (293FT/DSP2) are fused. The protocol of the DSP assay was previously described (28). Briefly, pCA7 expression plasmids encoding the WT H protein, one of MeV F proteins used in this study, with a pCA7 plasmid encoding a host protein or none, were transfected into cocultured 293FT/DSP1 and 293FT/DSP2 cells in 24-well plates using Lipofectamine LTX (Thermo Fisher Scientific). The *Renilla* luciferase activity was analyzed 24 hours after transfection using the *Renilla* luciferase assay system (Promega). Data represent three biological replicates, where each replicate corresponds to an independent well containing separately cultured cells. Luciferase activity was measured once per well, without additional technical replicates.

To evaluate the *trans*-acting functions of host molecules in fusion triggering, 293FT/DSP1 cells were transfected with pCA7 plasmids encoding CADM1, CADM2, CADM3, CADM4, nectin-3, or CRTAM or with pCA7 alone as a control. The cells were mixed with 293FT/DSP2 cells transfected with pCA7 plasmids respectively encoding the WT H and F(G264E/T461I) proteins. To evaluate the *cis*-acting functions, 293FT/DSP1 cells were transfected with the empty pCA7 plasmid. The cells were mixed with 293FT/DSP2 cells transfected with pCA7 plasmids respectively encoding the WT H protein, the F(G264E/T461I) protein, and one of host molecules. The *Renilla* luciferase activity was analyzed using the *Renilla* luciferase assay system (Promega) 24 hours after the cells were mixed.

### Cell surface biotinylation assay

Following procedures previously described (12), subconfluent monolayers of 293FT and CHO cells cultured on 12-well plates were transfected with pCA7 encoding one of MeV F proteins [Flag-tagged WT F, F(G264E), F(T461I), F(G264E/T461I), F(F375S), F(I446N), F(F375S/I446N), F(F375S/T461I), F(I446N/T461I), F(F375S/I446N/T461I), F_Patient B, F_OSA-3, and F_OSA-3-N446I] with pCA7 encoding E-cadherin (a cell surface protein control) using Lipofectamine LTX. At 24 hours after transfection, cells were washed with phosphate-buffered saline (PBS) and then incubated with 200 µL of the biotin reagent solution (2-mM EZ-Link N-hydroxysulfosuccinimide [sulfo-NHS]-biotin [Thermo Scientific] in PBS) for 30 min at 4°C. After the cells were incubated with PBS containing 50-mM ammonium chloride for quenching for 10 min at 4°C, they were lysed in 200 µL of the immunoprecipitation (IP) lysis buffer (Thermo Fisher Scientific) containing the protease inhibitor. The lysates were cleared by centrifugation for 30 min at 17,400 × *g* and 4°C. Then, 50 µL of each supernatant was mixed with an equal volume of 2 × sodium dodecyl sulfate (SDS) loading buffer (125-mM Tris-HCl [pH 6.8], 10% 2-mercaptoethanol, 4% SDS, 0.1% bromophenol blue, and 20% glycerol), boiled for 5 min, and stored at −20°C as the cell lysate samples. The rest of the supernatant was incubated with avidin-agarose beads for 3 hours at 4°C. The samples were centrifuged and washed three times with IP lysis buffer. Pellets were mixed with 30 µL of 2 × SDS loading buffer, boiled for 5 min, and stored at −20°C as biotinylated cell surface protein samples.

### Western blotting

Following procedures previously described (12), proteins in samples were separated by SDS-polyacrylamide gel electrophoresis and then blotted onto polyvinylidene difluoride membranes (Hybond-P, Amersham Biosciences). The membranes were incubated with primary antibodies (Abs) for 1 hour. Rabbit polyclonal Abs against Flag tag (F7425; Sigma-Aldrich) and mouse monoclonal Abs against E-cadherin (clone SHE78-7; TaKaRa Bio) and β-actin (clone BA3R, BioVision) were used. The membranes were washed with tris-buffered saline containing 0.05% Tween 20 (TBS-T) and incubated with horseradish peroxidase-conjugated goat anti-rabbit or mouse immunoglobulin G (Bio-Rad) for more than 1 hour at room temperature. After being washed with TBS-T, the membranes were treated with Chemi-Lumi One Super (Nacalai Tesque), and chemiluminescent signals were detected and imaged using a VersaDoc 5000 imager (Bio-Rad).

### Fusion-mediated spread assay

Mouse primary neurons cultured in 6-well plates were transfected with pCA7 plasmids respectively encoding the WT H protein, one of MeV F proteins (WT-F, F(F375S), F(I446N), F(T461I), F(F375S/I446N), F(F375S/T461I), F(I446N/T461I), F(F375S/I446N/T461I), F_Patient B, F_OSA-3, or F_OSA-3-N446I) , and mNeonGreen using NeuroMag Transfection Reagent (OZ Biosciences). The cell culture plates were placed on a magnetic plate for 20 minutes, and the cells were observed under a fluorescence microscope 24–30 hours after transfection. Fluorescence images of six microscopic fields per sample were randomly captured, and the areas of neurons expressing mNeonGreen were quantified by using ImageJ (36).

### Statistical analysis

All statistical analysis was performed using GraphPad Prism 10.2.0.

## Data Availability

The data sets generated and/or analyzed during the current study are available from the corresponding author on reasonable request. Source data for figures are available in Figshare at DOI: 10.6084/m9.figshare.13566947.
